# Organizational readiness to implement the Serious Illness Care Program in hospital settings in Sweden

**DOI:** 10.1186/s12913-022-07923-5

**Published:** 2022-04-22

**Authors:** Sofia Andersson, Anna Sandgren

**Affiliations:** Center for Collaborative Palliative Care, Department of Health and Caring Sciences, Linnaeus University, Växjö, Sweden

**Keywords:** Conversation, Implementation, Organization readiness for change, Palliative care, Qualitative, Serious illness, Serious illness program

## Abstract

**Background:**

The Serious Illness Care Program (SICP) is a model developed for structured communication, identifying patients, and training physicians to use a structured guide for conversations with patients and family members. However, there is a lack of knowledge regarding the sustainable implementation of this conversation model. Therefore, the aim of this study was to identify barriers and enablers during the implementation of the SICP in hospital settings.

**Methods:**

The SICP was implemented at 20 units in two hospitals in Sweden. During the implementation process, seven individual interviews and two group interviews were conducted with seven facilitators (five physicians, one behavioral therapist, and one administrator). Data were analyzed using qualitative content analysis, first inductively, and then deductively using the organizational readiness for change as a theoretical framework.

**Result:**

The analysis resulted in three factors acting as enablers and eight factors acting as enablers and/or barriers during the implementation of the SICP. The three factors considered as enablers were *preliminaries*, *identifying patients, and facilitator’s role*. The eight factors considered as enablers and/or barriers were *broad implementation*, *leadership, time, confidence*, *building foundation*, *motivation to work change*, *motivation for training in serious illness conversations*, and *attitudes*.

**Conclusion:**

This study indicates limited readiness to implement the SICP in hospital settings due to considerable variation in organizational contextual factors, change efficacy, and change commitment. The identified enablers and barriers for implementation of the SICP could guide and support future implementations to be sustainable over time.

**Supplementary Information:**

The online version contains supplementary material available at 10.1186/s12913-022-07923-5.

## Background

Early introduction to palliative care in the disease trajectory can improve the quality of care for patients and their family members [1–3]. It has been shown that patients with serious illnesses want to be involved in the decision-making of their care and care goals [4, 5]. Earlier studies have shown that end-of-life conversations are important for patients and family members and are associated with better symptom relief [6, 7]. It has been shown that if residents in nursing homes and family members participate in end-of-life discussions with physicians there is a higher probability of symptom relief for pain and anxiety [7, 8]. Another study has shown that if patients can converse with their physicians about their goals and care they are also more likely to receive the care they want [9]. Recognition of patients’ perspectives by healthcare providers is valuable for enabling better involvement of patients, however, these conversations are often received too late [1].

It has been found that physicians report barriers for lack of communication and fear of taking away hope in patients [10, 11]. In addition, nurses have reported barriers in communication about end-of-life care and prognosis in patients with heart failure [12]. A review has shown evidence that training in communication skills can improve care as professionals reported self-efficacy, confidence, competence, and communication skills [13]. Another review reported that structured early discussions of serious illness care goals were associated with beneficial outcomes for patients [14].

The Serious Illness Care Program (SICP) is a model developed for structured communication, identifying patients, and training physicians to use a structured guide for conversations with patients and family members. The program includes preparing physicians for these conversations, documenting these conversations in medical records, and sharing this information with other care professionals [15]. The model aims to offer the patient and family members the opportunity to talk about their situation, goals, and priorities. The model has been used for example in the United States and Great Britain [16–23]. Earlier studies showed that implementation of the SICP resulted in earlier and better documentation. Furthermore, the patients received more detailed information about their illness and were able to talk about their goals and preferences [24]. Geerse et al. [18] showed that patients initiated conversations about emotional queries more often, but clinicians had difficulty responding to these types of emotional conversations. Further, clinicians were quick to offer positive affirmations and reassured that patients would be kept comfortable. It has also been found that the quality of documented serious illness conversations (SIC) after implementation of the SICP was significantly higher than during usual care, with the clinicians and patients more often discussing patients’ values and goals and understanding of prognosis and illness [25]. Research on the use of the SICP in palliative care has increased in recent years, with positive outcomes for both patients and family members as well as for health care professionals. However, research on the sustainability of the SICP implementation needs to be further explored. In a Swedish project, the SICP was culturally adjusted and implemented at 20 units in two hospitals, with a focus on specialist physicians, in southern Sweden. This study explores organizational readiness to implement the SICP. The aim was to identify barriers and enablers during the implementation of the SICP in hospital settings.

## Methods

In this study a qualitative design was used, and both individual and focus group interviews were conducted. The consolidated criteria for reporting qualitative research (COREQ) were used to report the study [26].

### Study setting

The SICP was implemented in two acute-care hospitals in a region in southern Sweden, serving almost 200,000 inhabitants.

### Study participants

The inclusion criteria for this study were persons whose roles were facilitators during the entire implementation of the SICP. The facilitators (*n* = 7) received an information letter about the study from the project leader (AS) and were invited to participate. All seven facilitators accepted and were included: five physicians, one behavioral therapist, and one administrator; four women and three men.

### Serious illness care program (SICP)

The SICP is a multicomponent and structured communication intervention that intends to facilitate conversations with patients with serious illnesses [14]. The decision to implement the SICP originated from the leaders of the hospitals. The SICP was contextually adapted to the Swedish context, for example through removing questions about living wills or advance directives from the patient preparation document. Instead of 3 hours of training for the physicians, as in the original model, they received 8 hours of training with professional actors. The extended training included how to reply and handle patients’ emotional feelings during the conversations. Another adaptation was that the nurses made a follow-up phone call 2 weeks after the conversation to inquire about the patients’ experiences of their conversations with the physicians. This was considered important since this was a new way of working. The serious illness conversation guide was translated into Swedish and adjusted to suit the Swedish context.

The SICP was implemented in 20 units at two hospitals. In total, 106 specialist physicians received one-day training in conversation skills based on the serious illness conversation guide (34 medicine, 20 surgery, 11 pediatric care, 9 ophthalmology, 7 infection, 7 ear nose throat, 6 palliative medicine, 5 anesthesia, 3 oncology, 3 gynecology, 1 orthopedic). During the training day, they practiced together with actors and received coaching from the training leaders. After the training, the facilitators had meetings with the units to discuss how to identify patients who would benefit from SIC. The surprise question of “Would you be surprised if this patient died within the next year?” [27] could be used to identify patients. When patients were identified, the nurses provided both verbal and written information about the possibility of having an extended conversation with the physician, with a focus on their own goals and priorities. If the patients wanted these conversations, the nurses booked the appointments. During conversations with patients, the physicians used the conversation guide, and conversations were documented in the medical record. After 2 weeks, the nurses phoned the patients and asked them about their experiences with the conversations. During the entire implementation process the facilitators met with the managers and physicians several times to support and remind them to continue with the SIC.

### Data collection

During the implementation process, both individual and focus group interviews were conducted with facilitators. Seven individual interviews were conducted in June 2019 (EH and SMJ), one focus group interview in November 2019 (first author), and one later in May 2020 (first author). Focus group interviews were conducted to explore the changes and facilitators’ experiences of the implementation over time. All interviews were conducted in person except for the last one, which was held on Zoom due to the COVID-19 pandemic. The individual interviews lasted between 20 and 62 min, and the focus group interviews lasted 63 and 71 min, respectively.

The semi-structured interview guide (Additional file 1) included questions about the implementation of the SICP and its different parts. The interviews started with the opening question: “Can you describe how the implementation of serious illness conversations is going?”. Then, questions were asked about the benefits and challenges of the new way of working, their view of the managers’ and physicians’ readiness for change, and their motivation for implementing the SICP.

### Data analyses

The analysis was first conducted with an inductive approach using conventional content analysis, according to Hsieh and Shannon [28]. The authors read all the interviews repeatedly to obtain a sense of the whole. Then, the first author thoroughly read all interviews and highlighted the exact words from the text that appeared to capture key thoughts or concepts on how the SICP was implemented and its process. The authors then discussed key thoughts, approached the text again, and the first impressions, thoughts, and the first analysis were written. The process continued with labeling for the codes to produce more reflective labels than the keynotes and become the initial coding scheme. After that, the codes were sorted into categories depending on differences, similarities, and content. During the inductive analysis process, the data were first analyzed with a time perspective, but no clear differences were found. Therefore, we decided to consider the data as one data set in the continuation of the analysis. During every step of the analysis process the authors held meetings and discussed codes and categories until consensus was reached.

In the next step, a deductive approach was used with organizational readiness for change (ORC) as a theoretical framework to analyze the data. ORC includes *contextual factors*, *change efficacy*, and *change commitment* and is not only a multilevel construct but also a multi-faceted one. Specifically, organizational readiness refers to the organizational members’ commitment to change and change efficacy in implementing organizational change [29].

*Contextual factors* include the organization’s preparedness and readiness. Leadership is also important for members’ willingness and ability to make changes. The contextual conditions could increase or decrease the change valence associated with a specific organizational change, depending on whether the change effort fits or conflicts with cultural values. Organizational policies, procedures, resource availability, or earlier experiences can positively or negatively affect organizational members’ appraisals. *Change efficacy* means that the participants believe in their capacity to make a change. Change efficacy is higher when people share a sense of confidence that they can collectively implement a complex organizational change. *Change commitment* means that the more members value the change, the more they will want to implement the change, or the more resolve they will feel to engage in the courses of action involved in change implementation [29].

In this step, the categories from the first analysis were compared and sorted in contrast to the three constructs of the ORC. Pattern matching was performed using the three components of the ORC theory and matching them with categories. In this step, the authors held several meetings, discussing the different categories in relation to the three constructs until consensus was reached.

### Ethical considerations

Ethical approval was obtained from the Swedish Ethical Review Authority (reference number: 2018/540–31). Permission to conduct the study was obtained from the heads of the hospitals. Written and verbal information was provided to all participants along with the information that participation was voluntary and that the participants could withdraw without giving any explanation. Written informed consent was obtained from all participants.

## Results

The analysis resulted in three factors (i.e., sub-categories) acting as enablers (E) and eight factors acting as enablers and/or barriers (E/B) during the implementation of the SICP (Table 1). Quotes illustrate findings from individual interviews (I1-I7) and focus group interviews (FG1, FG2).

**Table 1:** Overview of the categories/constructs in ORC, the subcategories/factors, and their explanations

Category in ORC/ Construct	Subcategory/Factor	Explanation
Contextual factors	Preliminaries (E)	The preliminaries are described as an enabler and include meetings with various regional representatives, adjustments of the SICP into the Swedish context, and a pilot study.
	Broad Implementation (E/B)	Broad implementation is described both as an enabler and a barrier and is related to introducing the SICP in several units at the same time. Facilitators were unable to maintain omnipresence for every unit.
	Leadership (E/B)	Leadership is described both as an enabler and a barrier. It was an important part in the implementation process and to create possibilities for the initiation of implementation. If the manager does not encourage the physicians, it is described as a barrier.
	Time (E/B)	Time is described as both an enabler and barrier. It could be timesaving as well as time consuming.
	Confidence (E/B)	Confidence and/or lack of confidence is also described as an enabler and a barrier for the physicians. Physicians seem to have more confidence to have these conversations after the training, A barrier was that the physicians sometimes seemed unconfident, uncomfortable, and afraid to take away the patients’ hope.
Change efficacy	Building foundation (E/B)	The building foundation is both an enabler and a barrier. This is done in several steps over time. Facilitators held several meetings with managers, but there was a lack of resources to support all units.
	Identification (E)	Identification of patients in need of SIC is described as an enabler for the implementation process. It is a key issue for implementation.
	Motivation for work of change (E/B)	Motivation for work of change is described as both an enabler and a barrier. Motivation to implement the SICP.
Change commitment	Motivation for training in SIC (E/B)	Motivation to undergo education about SIC was described as both an enabler and a barrier.
	Attitudes (E/B)	Physicians’ attitudes and their own capabilities in SIC are described as both an enabler and a barrier.
	Facilitator’s role (E)	Facilitator’s role is to support the units and was described as an important enabler to make the implementation possible.

*ORC* The organizational readiness for change; *E* enablers, *E/B* enablers and/or barriers, *SICP* Serious Illness Care Program, *SIC* Serious Illness Conversation

### Contextual factors

Contextual factors include *preliminaries* (E), *broad implementation* (E/B), *leadership* (E/B), and *time* (E/B).

Before implementation began, *preliminaries* were made. Preliminaries means preparation before the start of the SICP, such as prepared guides, planning, and a pilot project. This was regarded as an enabler and included, for example, writing project plans and planning for how and when different steps could be started. The different documents and guides were translated into Swedish with a cross-cultural adaptation into the Swedish context (not scientifically done). A group of patients and family members were involved in the planning and translation of guidelines, which was described as an important aspect by the facilitators. In the Swedish-adjusted model, it was decided that nurses should ask the patients if they wanted a conversation with a physician and then phone them 2 weeks after the conversation to ask how they were doing and about their experience of the conversation. During the *preliminaries*, a pilot study was conducted in 4 units and received good evaluations; however, it was not scientifically evaluated. Adaptations were made in the conversation guide to be used in conversations with patients with loss of function. Facilitators described that the *preliminaries* took time and resources.

*A: A key factor was that we had a group of patients and family members from the beginning, which looked at the documents that were allowed to express their views of the phrasing that we have used, and giving us suggestions. (FG1)*

The *broad implementation* was described as an enabler because it created possibilities to include several units at the two hospitals simultaneously and allowed the specialist physicians at the units to receive training at the same time. However, the *broad implementation* was also seen as a barrier because it was difficult for the facilitators to focus and support the units simultaneously due to lack of resources. The fact that the units cared for patients with different diagnoses and prognoses was considered as a barrier for the implementation, since the implementation strategies needed to be adjusted to suit the different contexts, which was challenging.

*“All 20 units had different needs, and different patient groups; therefore one must modify the model to adapt for their different needs” (I1)*

Clear *leadership* was an enabler and an essential factor. Most managers were positive towards the implementation. If the manager had an active *leadership style*, implementation was considered more successful. The managers needed to take more explicit *leadership* roles and describe how they could create possibilities for implementation. Some managers lacked this commitment and did not understand their role in the implementation, so managers needed to be more involved with explicit responsibilities. Suggestions for future implementations were to use a checklist or a contract with managers before the implementation started.

*A: That is a point of improvement that we should have focused on even more, especially explicit in what we expect from the manager, now it is yours, meaning our role is this and your role is that, and we cannot perform our role if you do not perform yours. I think that we could have been much clearer and almost such that they should sign some form of contract or something similar. We will be glad to educate your coworkers under these circumstances. (FG1)*

*Time* was described as both an enabler and a barrier. The implementation could save *time* for both the physicians and the organization because both the patients and the family members received the same information at the same time. However, *time* was described as the most significant barrier for implementation, even for physicians who prioritized these conversations. The SIC could be an extra conversation for the physicians in their already overbooked day, and then the physicians did not have *time* for regular return visits. The facilitators also described that supporting all the units was time consuming and that they lacked the time to do so.

*I think that is the biggest challenge in a pretty hard-pressed organization, there is no space in the system, we have too little time for physicians, and there is a lack of specialists (I6).*

### Change efficacy

Change efficacy includes *confidence* (E/B), *building foundation* (E/B), and *identification* (E).

Facilitators considered that the implementation of the SICP gave the physicians more *confidence* to have conversations with patients and to meet patients’ reactions. They also mentioned that the physicians seemed to have more self-efficacy in their ability to have this conversation after the training, through learning new skills and communication tools. If the physicians were prepared, they could feel more self-efficacy in meeting ambivalent patients, and they could provide comfort. However, a barrier was that the physicians sometimes seemed unconfident and uncomfortable, and they were afraid that the patients would feel hurt by the conversations or that it would take away the patients’ hope. Another barrier was that some physicians were unconfident and *uncomfortable* because they did not have any training for these kinds of conversations. There were also some misunderstandings about feeling forced to talk about prognosis during the conversations, instead of offering a discussion about prognosis to the patients.

*“The thing is that knowledge is always good to have and one becomes safe in one’s work with having these difficult conversations when one gets tools” (I5)*

*“Then we take away the hope,” that has been said several times. “Should we really talk about prognosis? Should we start this conversation? Are we doing good?” (I1)*

During *the building foundation*, the facilitators held several meetings with managers. If the managers were engaged and saw the need for change and the need for increased competence, it was considered an enabler for implementation in that unit. However, *the building foundation* in several units was also considered as a barrier because of the lack of resources to support all the units, and because the units were different. The facilitators, therefore, decided that they needed to focus on the units in which the managers were more involved and motivated. Some managers were satisfied that the physicians had received the training and it was regarded as “good enough”. This could be seen as a barrier to implementing the entire model and changing the working method.

*“In retrospect, it is not enough with one information meeting, rather it would be necessary to repeat this several times since they could have had some sort of home assignment to see what needs we actually have and what amount of time we are prepared to set aside for this” (FG2)*

*Identifying* patients in need of SIC was an enabler and an essential factor in the implementation process. Ongoing care becomes easier when the patients have been *identified* and they are offered conversations so that their wishes are known. When *identifying* the patients, the “surprise question” could be used. However, when nurses and physicians did not agree with the patients’ prognosis it could be difficult to *identify* the “right” patients. The nurses were described as key persons in the *identification* process because they often had more contact with the patients and knew their current situation.

*“In some way, it is one of the key factors in the care flow, to ensure a care-specific flow of activities you must recognize the patient.” (I2)*

### Change commitment

Change commitment includes *motivation to work change* (E/B), *motivation for training in* SIC (E/B), *attitudes* (E/B), and *facilitators´ roles* (E).

The facilitators described that the *motivation for work change* within the unit was an important factor for the success of the change. Facilitators met both physicians who were motivated and not motivated to change. The facilitators had the impression that the nurses were sometimes more motivated than the physicians. An important factor that increased their motivation was their first SIC. Using good examples from units that have started to use these conversations increased the motivation of those who had not yet started. It was important that the physicians had the possibility to influence which time of the day the conversations were booked. If physicians were motivated, the implementation turned out to be more sustainable. If the physicians at one unit showed low motivation, the facilitators decided to return to this unit later and instead focused their mentoring on physicians with higher motivation. This decision was made due to the lack of time and resources for the facilitators.

*1: “And then where should their motivation come from to try to start working in a new way, that we think is so important*

*2: Yes, exactly*

*1: They might understand the need for it, but it is drowned out by the workday’s usual activities, among other things” (FG1)*

If the physicians were *motivated to the training day*, it was more common for the implementation to be successful. If all physicians at one unit participated in the same training day and that they had already scheduled a SIC with a patient after the training day, it increased the motivation for the training day. The facilitators described that some physicians were not *motivated* and were not informed about why they had to participate. Some physicians seemed nervous, and they were not prepared for what was going to happen on the training day. A barrier was that it was uncomfortable to stand in front of colleagues, and it was not voluntary for the physicians to attend the training day. Another obstacle that emerged was that several physicians had limited communication skills. There was also a difference between training on how to conduct these conversations and then actually conducting them in practice. Another barrier for implementation was that only specialist physicians were involved, who seemed less *motivated for training* compared to the younger physicians.

*“Yes, (training-) days are totally different depending on if the participants have already understood what it is about and accepted the model. Because then they come to the training day and want to test it, see how it works, and they get to practice the conversation method” (I7).*

The facilitators believed that there was a positive *attitude* among some physicians, which was favorable for the implementation. Younger physicians and nurses were described as having a more positive *attitude* toward new ways of working, while other physicians were more ambivalent about who they thought should hold the SIC. Some physicians had positive attitudes towards the SICP since they saw it as a possibility to integrate palliative care into regular care at an early stage in the patient’s disease trajectory. However, it also appeared that some physicians had a negative *attitude* toward the implementation; they did not see that the patients needed SIC.

*Some of them were uninterested. Some have raised questions, sometimes being almost aggressive. Then again, some of them have been really kind, asking more questions.* "*What do you think about this?" They are interested in the research results… and some have outright said: ‘This is exactly what is needed right here’ (I2)*

The *facilitators’ role* was important for the change of work, especially at the beginning of the implementation. To provide the right support based on the unit’s needs, the facilitators needed an understanding and closeness to each unit. In the beginning, there were mentors to coach the physicians, but since only a few SICs were held this specific support was ended. *Facilitators’ roles* require continuity. At the start of the implementation the facilitators held regular meetings with the physicians and nurses, but later they sent reminders through email. Being a facilitator was considered challenging, and they described a lack of resources and that it had taken a lot of energy. However, they helped and supported each other. They would have needed more persons for the *facilitator’s role* due to the broad implementation. The COVID-19 pandemic and its impact on healthcare made the *role of the facilitators* more challenging. To make the implementation sustainable, there needs to be adequate resources since it takes time to change the working way and the professionals’ attitudes.

*2: “And that we should, like, have a larger set of people that work with the implementation.*

*1: that we should be more*

*2: yes, and a more clear structure of what we should do.*

*1: and not attempt to do it along everything else that we do?*

*2: No, precisely… we already knew all that but then one jumped into it.” (FG1)*

## Discussion

This study aimed to explore organizational readiness to implement the SICP in hospital settings from the perspective of facilitators. When using ORC with its three overarching categories as a theoretical framework [29] in our analysis, we identified 11 different factors, of which three acted as enablers and eight acted as both enablers and barriers during the implementation of the SICP.

Preliminaries, identification of patients, and the facilitator’s role were factors which acted as enablers for the implementation. The preliminaries were all important, but especially in the pilot study, which was conducted before starting the broader implementation. The role of pilot studies has been shown in an earlier study by Pasricha et al. [30], who found that it relieved the providers’ anxiety and enhanced their professional satisfaction.

Our results showed that although identifying the “right” patients for SIC was very important, it was difficult at the same time. Although the World Health Organization’s definition of palliative care includes patients and family members who live with a life-threatening illness [3], the different perceptions of which patients have palliative care needs were found to be challenging in our study. This issue has been described earlier as a blurred conceptual understanding of palliative care [31], which creates challenges in identifying patients [17, 20]. In our study as well as in other studies, the surprise question of “Would you be surprised if this patient died in the next 12 months?” has been used to identify patients who need SIC [17, 20, 32]. Our study shows that different diagnoses, such as cancer and heart failure, could make it more challenging to identify patients in need of SIC. This was also shown in another study [33], in which they found that by using the surprise question, only six of 23 patients were identified. They also found issues with the feasibility of different prognoses and situations [33]. Further research is needed to evaluate if the surprise question identifies patients with specific diagnoses for SIC better than others.

However, several different ways to identify patients for SICP beyond the surprise question have been used, such as registries or algorithms and disease-specific criteria [17]. Another method for identification could be the use of clinical tools. The Supportive and Palliative Care Indicators Tool (SPICT) has been developed and evaluated to identify patients who are at risk of deterioration. When using SPICT, it gives patients the opportunity to talk about their health and what matters to them. The use of SPICT has also been shown to provide patients with a better quality of life and to involve family members [34]. SPICT has been translated into Swedish with a cross-cultural adaptation, and it has been shown to be well designed with all the indicators for palliative care [35]. Therefore, the SPICT instrument may be a better instrument for identifying patients with serious illnesses. Research is needed to evaluate whether SPICT can be an option for the identification of patients with serious illness.

The facilitator’s role, with ongoing coaching, was an important factor for successful implementation. Alexander Cole et al. [36] described that the virtual coaching of physicians who had already received communication skills training in SIC resulted in an increase in documented SIC. The results also showed that it was an opportunity to reflect on the usefulness of prior skills training, electronic health documentation, sharing experiences and receiving expert support from palliative care coaches [36]. Williams et al. [37] indicated that a key factor for identifying challenges and solutions is having structured stakeholder feedback mechanisms [37]. Another study reported that organizational changes were more likely to be successful if health care professionals have the opportunity to influence the change, are prepared for the change, and recognize the value of the change [38]. The same result was observed by Nilsen et al. [39], but also a passive and active resistance to change. Another study that implemented knowledge-based palliative care in nursing homes showed that if the care professional was involved in the process and that the changes were well grounded, it was favorable for implementation [40]. Feedback from facilitators to care professionals about how the implementation functions seems essential for sustainability and successful implementation. In our study, the facilitators played an important role in supporting and coaching professionals during the implementation. A key factor was that the facilitators had knowledge about the context and were nearby to meet the physicians and to remind them.

Furthermore, we identified eight factors that acted as enablers and/or barriers for the implementation of the SICP in hospital settings. One barrier for implementation was the physicians’ fear of taking away patients’ hope when talking about what might happen. However, a recent study showed that patients felt SIC helped them to reflect on their prognosis and goals of care, and did not decrease their hope or provoke anxiety [41]. Another study described that the conversations were useful, and although they did not find any differences in patients’ experiences of peacefulness or hopefulness, they found that the patients experienced better planning for future care with a focus on their personal priorities [22]. Patients have also reported that they increased their relationship with their physician, and almost half of the residents reported increased hopefulness [42]. Family members want to discuss prognosis with the physicians [43]. Family members have described that their understanding of their loved ones in critical care settings improved, and that it created a sense of control of the medical decision [30]. Sharing difficult medical decisions is complicated, and clinicians must respond to emotions [44]. One way to make the physicians more comfortable could be to give feedback to the physicians about patient experiences after the conversations.

Motivation for work of change acted as both an enabler and a barrier. Not all physicians were motivated, and some thought it was not their responsibility to have these conversations with the patients. However, nurses who worked closer to the patients were more likely to implement the SICP. The importance of identifying clear roles and responsibilities for care professionals has been highlighted in earlier implementations of the SICP [17]. Our study found that the physicians’ motivation for the training day was both an enabler and a barrier. Some physicians were motivated, while others had not been informed about the purpose of the training day. Some physicians were nervous and uncomfortable to train in front of colleagues. This is in line with another study reporting that the training day could be uncomfortable for physicians; however, it plays an integral role in learning about how to have SIC [45].

The building foundation and the facilitators’ role during implementation are important in the implementation of SICP. Organizational readiness in the building foundation is likely to be highest when organizational managers want to implement organizational change, feel confident, and support physicians. Weiner [29] suggested that if professionals see the need, the benefits, and the value of change, they are more willing to implement the SICP [29].

In our study, the facilitators described the nurses as favorable to the implementation, and we argue that one way to make the implementation of SICP more sustainable would involve nurses more in the SICP. Further implementation should focus on team collaboration, with SIC training for physicians as well as for nurses. Lakin et al. [17] described that interprofessional teamwork enables SICP work, and nurses were also described as a key factor in the implementation process. Further research is needed with a focus on teamwork as well as the specific roles that different care professionals have in the SICP. Another important research area is the implementation of SICP with a focus on sustainability over time.

### Methodological considerations

This study had both strengths and limitations. One strength was that data collection was done with the facilitators several times during the implementation. We also combined individual and group interviews to ensure richer and more comprehensive content over time. Even if the result does not reflect differences over time, the longitudinal data collection captures the facilitators’ experiences of the implementation as it happened, which otherwise would have been lost. A limitation could be that the participants felt an expectation from the others in the focus group to focus on the positive aspects of the implementation, but in the interviews both positive and negative issues were raised. The focus group allowed interactions between the participants, which enabled opportunities for argument or agreement with others in the group [46]. The data analysis was first performed inductively and then deductively using the ORC with its three overarching categories [29] as a framework for the analysis. The content and structure of the ORC offered a theoretical explanation for the determinants of implementing SICP and was a valuable way to explain the enablers and barriers when implementing the model. However, using ORC as a framework for analysis could also be seen as a risk for excluding relevant data. This risk is regarded as low because we first analyzed the data inductively, and it was not until the second phase in the analysis, we applied the ORC framework and identified factors as enablers and barriers. However, we do not claim that all factors relevant to the implementation of SICP have been identified, and further studies may identify other possible determinants. The setting, data collection, and analysis were described as thoroughly as possible to ensure credibility. In the results, the different factors were also verified with quotations from both individual interviews and focus group interviews.

## Conclusion

This study indicates a limited readiness to implement SICP in the hospital setting due to considerable variation in organizational contextual factors, change efficacy, and change commitment. Both enablers and barriers to implementing the SICP were identified. When implementing the SICP in the future, the enablers and barriers identified in this study need to be considered in order to reach a sustainable new way of working in an organization.

## Supplementary Information

**Additional file 1.** The semi-structured interview guide.

## Data Availability

The datasets used and/or analyzed during the current study are available from the corresponding author upon reasonable request.

## Abbreviations

SICP: Serious Illness Care Program
SIC: Serious Illness Conversation
