# Supplementary material for: Organizational readiness to implement the Serious Illness Care Program in hospital settings in Sweden
Source: BMC Health Serv Res. 2022 Apr 22;22:539. doi: 10.1186/s12913-022-07923-5 (PMC9026003; doi:10.1186/s12913-022-07923-5)
Supplement: Supplementary file 1 — Additional file 1. The semi-structured interview guide. [file 12913_2022_7923_MOESM1_ESM.docx]

**Additional File**

*The semi-structured interview guide (Additional File 1)*

Can you describe where you are with implementing the Kronoberg model?

Can you describe the implementation process of the Kronoberg model?

- How do you see the anchoring of the Kronoberg model in your organization?
- How is the readiness in your organization for the future?
- Broad implementation – facilitators- hinders?
- The introduction of working methods at the clinic level? The introduction of working methods at the clinic level?
- What attitude would you say that the staff and management have to this project's change of duty/care? Over all?
- What values and attitudes of staff can hinder or enable the introduction of a new way of working?
- Have values and attitudes changed among staff?

What are the prerequisites in the implementation of this approach? Over all?

What do you think hinders the implementation of this approach? Over all?

Can you identify any key events that have been crucial in implementing this new way of working?

- Facilitators/hinder key events
- How do you think that event has affected the outcome?

From the beginning, there was an idea with the model. How do you see it has changed? Can you tell me what you thought about the model and what it is like now?

- What changes have taken place?
- Why have they happened?

What was it like before the introduction of the model? What's the difference now? What do you think it will look like in the future, i.e. the goal of the new way of working?

What is the biggest benefit of this new approach?
